# Fatty Acid Profile of Postmenopausal Women Receiving, and Not Receiving, Hormone Replacement Therapy

**DOI:** 10.3390/ijerph16214273

**Published:** 2019-11-04

**Authors:** Anna Maria Cybulska, Karolina Skonieczna-Żydecka, Arleta Drozd, Kamila Rachubińska, Jolanta Pawlik, Ewa Stachowska, Anna Jurczak, Elżbieta Grochans

**Affiliations:** 1 Department of Nursing, Pomeranian Medical University in Szczecin, 71-210 Szczecin, Poland; anna.cybulska@pum.edu.pl (A.M.C.); grochans@pum.edu.pl (E.G.); 2 Department of Human Nutrition and Metabolomics, Pomeranian Medical University in Szczecin, 71-460 Szczecin, Poland; arleta.drozd@gmail.com (A.D.); ewa.stachowska@pum.edu.pl (E.S.); 3 Student Research Association, Department of Nursing, Pomeranian Medical University in Szczecin, 71-210 Szczecin, Poland; k.rachubinska@gmail.com; 4 Institut für Nephrologie und Dialyse Salem-Spital, Hirslanden Bern AG, 3000 Bern, Switzerland; jola.pum@wp.pl; 5 Department of Specialized Nursing, Pomeranian Medical University in Szczecin, 71-210 Szczecin, Poland; anna.jurczak@pum.edu.pl

**Keywords:** menopause, menopausal hormonal therapy, fatty acid

## Abstract

Menopause, the permanent cessation of the menstrual cycle, marks the end of a woman’s reproductive lifespan. Menopausal hormonal therapy (MHT) can potentially skew the fatty acid profile increasing the risk for developing metabolic diseases and disorders of skeletal, gastrointestinal, and nervous systems. The aim of this study was to investigate the fatty acid profile of postmenopausal women receiving, and not receiving, hormone replacement therapy. A total of 156 healthy women with a mean age of 60 participated in this cross-sectional study. Gas chromatography with an Agilent Technologies 7890A GC system was used to determine fatty acid content. Statistical analysis was conducted using R software, version 3.4.1. Women receiving MHT had significantly higher (*p* < 0.05) concentrations of C14:0 and C16:0. MHT was found to be associated with a tendency (*p* = 0.053) to diminish concentrations of C18:1n-9, C20:4, and all unsaturated fatty acids (*p* < 0.05). The longer MHT was used, the higher the concentration of C24:1 (*p* = 0.04) and the lower the concentration of C18:2n-6 (*p* = 0.03).

## 1. Introduction

Menopause usually appears between the ages of 40 and 50 years and typically cannot be entirely distinguished from the symptoms of the aging process. Menopause is associated with significant changes in a woman’s life in physiological, psychological, social, and cultural spheres. According to the World Health Organization’s 1996 definition, menopause is the final normal menstruation in a woman’s life, resulting from a complete loss of ovarian follicles, followed by a break of at least one year in menstrual bleeding [1,2,3]. The hormonal disruptions encompassing the climacteric, in particular, the deficiency of estrogen and progesterone, lead to a risk of developing metabolic diseases [4,5] and disorders of the skeletal [6], gastrointestinal, and nervous systems [7,8]. Abnormal hormone levels cause uncontrolled sadness, irritability, negative self-image, and even aggression [9,10].

Menopausal hormonal therapy (MHT) is used to treat the symptoms of menopause [11,12]. It can be administered in various formulations and modalities, and is available as systemic estrogen, estrogen-progestin, estrogen-bazedoxifene, progestin alone, and combined oral contraceptives [13,14]. The Women’s Health Initiative, the Million Women Study, has shown that the life-threatening effects of MHT outweigh its advantages [15], however, some observational studies independently carried out in recent years have failed to confirm these conclusions, and have sometimes contradicted them [16]. The North American Menopause Society concluded that hormone therapy still remains the most effective treatment of vasomotor symptoms and of the genitourinary syndromes of menopausal [17].

MHT should be initiated as soon as the first symptoms resulting from estrogen deficiency appear [18] and it has been determined that MHT should last longer than five years [19]. The risks associated with MHT vary depending on the type, dose, time of application, and administration route. It has been concluded that elevated doses of estrogen may induce cardiovascular events through alterations in thrombogenesis and vascular remodeling [20]. Generally, MHT is most beneficial for women younger than 60 and those within 10 years of the onset with menopause, with no contraindications [21]. The “timing hypothesis” exists which posits that younger women may not be at risk of adverse events following the use of MHT and may instead experience a survival advantage [22,23].

A fatty acid profile has a significant effect on health as it may contribute to the development of non-infectious diseases, such as overweight, obesity, metabolic syndrome, and nonalcoholic hepatic steatosis. In postmenopausal women, energy metabolism slows down and they are at a higher risk of lipid metabolic disorders [24], as well as cardiometabolic disorders associated with hormonal changes occurring in the body. The majority of studies have focused on the analysis of fatty acid metabolism, fatty acid (FA) levels mainly in patients with metabolic syndrome and diabetes [25], on pregnant and breastfeeding women [26], patients with cardiac diseases [27], and elderly people [28]. There are also many large-scale studies concerning both women and men without regard to age [29]. Considering the risk of lipid metabolic disorders observed in postmenopausal women, it seems important to assess the FA profile, which is responsible for the risk of non-infectious diet-related diseases, as well as the metabolism of FA and their levels in the blood.

There is a growing body of evidence that unsaturated fatty acids positively affect physiology. Among the four types of unsaturated acids, biological activity is specifically exhibited by the following two families: n-3 acids, such as α-linolenic acid (ALA) and n-6 acids, the precursor to which is linoleic acid (LA) [30]. It has been demonstrated that, during menopause, the conversion of fatty acids into long-chain polyunsaturated fatty acids (LCPUFAs), such as decosahexaenoic acid (DHA), is skewed, due to lower concentrations of estrogens and higher concentrations of testosterone [31]. This seems to be crucial, because ALA, the precursor of omega-3 LCPUFAs, is a component of cell membranes and plays a pivotal role in cell cycle mechanisms [32]. DHA is predominant in the membranes of nerve cells [33]. Metabolites of n-6 acids also have proinflammatory and prothrombotic activities, whereas metabolites of n-3 acids exert anti-inflammatory activity and inhibit platelet aggregation [34]. A high n-6/n-3 PUFA ratio has been shown to adversely affect health. Consequently, a skewed level of unsaturated fatty acids during menopause may, at least partly, explain the principal health concerns of menopause, which typically relate to cardiovascular health and mood [35,36]. It is worth noting that symptoms of these entities may be independently present in aging women, and thus during menopause [37]. Indeed, sex hormones have been demonstrated to be protective against cardiovascular disease, as estrogen has been shown to balance lipid profile [38], hemostasis [39,40], vasodilatation [41], and vascular remodeling [20].

Estrogen fluctuations and a skewed unsaturated fatty acid profile have been well documented in the menopausal period, and both may play roles in cardiovascular disease (CVD). On the other hand, at least a few studies have provided evidence that MHT can diminish these entities. In light of these facts, we found it reasonable to examine the fatty acid profiles of postmenopausal women receiving, and not receiving, MHT.

## 2. Materials and Methods

### 2.1. Study Group

The study involved 201 postmenopausal women (with established menopause) from the West Pomeranian Voivodeship, Poland. Recruitment was performed using information posters in public places and advertisements in local papers.

The inclusion criteria were:

- at least one year from the last menstruation;
- normal mammography results;
- normal cervical smear results;
- no clinically confirmed mental disease;
- normal blood pressure (less than 120 systolic and over 80 for diastolic);
- a normal diet without supplementation of fatty acids (including of omega 3), based on Polish cuisine.

The exclusion criteria were:

- less than one year from the last menstruation;
- abnormal mammography results;
- abnormal cervical smear results;
- diagnosis of a metabolic disorder (diabetes, thyroid disease);
- past or current cancer;
- diagnosis of a mental health problem;
- using MHT other than pills (for example hormone patches or suppositories).

From the group of 201 women who wished to take part in the study, we excluded those who did not meet the criteria. The remaining 156 women were divided into two groups: 107 women not using MHT, and 49 women taking oral estrogen-progestogen MHT in line with the gold standard (a low-dose therapy tailored to individual needs). All the MHT-using respondents had employed MHT following instructions from their gynecologists because their menopause was confirmed.

The research was carried out in accordance with the Helsinki declaration and with the approval of the Bioethical Commission of the Pomeranian Medical University in Szczecin (decision KB-0012/10/14 dated 3 February 2014).

### 2.2. Procedure

The research was carried out in several stages. After receiving written consent, persons with psychiatric disorders were withdrawn from the study group. The PRIME-MD Questionnaire was used for this purpose (Primary Care Evaluation of Mental Disorders Patient Health Questionnaire 9, PRIME-MD PHQ-9). This tool is used in primary care and includes symptoms from all criteria for diagnosing mental illness according to *Diagnostic and Statistical Manual of Mental Disorders*, Fourth Edition (DSM-IV); it incorporates a gradual scale of assessment of the severity of symptoms [42]. The most common diagnoses were depressive disorders in the case of lowered mood depression, anhedonia, and at least five symptoms in total during the two weeks preceding the study [43]. These conditions were clinically confirmed.

A questionnaire of our own devising was used; this contained a number of questions on demographic data and health, such as the date of last menstruation, the age of first menstruation, and the date of starting MHT.

In the next stage of the research, the weight and height of the subjects’ bodies were measured. For this purpose, we used a certified medical scale with an integrated SECA 711 increase gauge. A standardized procedure was following, yielding measurements with an accuracy of 0.1 kg and 0.1 cm. The body mass index (BMI) was calculated using the following formula: (body mass (kg) / height (m^2^). The women were classified as underweight (BMI < 18.5), normal weight (18.5–24.9), overweight (25.0–29.9), and class I obesity (30.0–34.9), class II obesity (35.0–39.9) and class III obesity (BMI ≥ 40.0) [44] based on the BMI. In order to calculate the waist-to-hip ratio (WHR), the abdominal circumference was measured along the axillary line, mid-distance between the rib arch and hip bone, using a measuring tape (Seca). According to WHO findings, the WHR is greater than 0.9 in obese women [45].

### 2.3. Sampling and Fatty Acid Analysis

The concentration of fatty acids was measured in whole blood. Blood samples were collected using the Vacutainer method. The patient had fasted to ensure a twelve-hour period between the last meal and blood collection. The samples were collected between 7:30 and 10:30 a.m. and were stored at −70 °C for analysis. Fatty acids were isolated using the Folch method (Folch, Osad, Stanley, 1957) [46]. Methylation of fatty acids was then performed, and the fatty acid content was determined using gas chromatography with an Agilent Technologies 7890A GC system. The compounds were separated using a Supelcowax 10-column Capillary GC Column (L × I.D. 15 m × 0.10 mm, df 0.10 μm) (Supelco, cat. no: 24343).

The temperature was held at 40 °C for 0.5 min, then increased at a rate of 25 °C/min to 195 °C, where it was held for 0 min; it was then raised at 3 °C/min to 205 °C, where it was held for 0 min before increasing further at 8 °C/min to 250 °C, to be held for 0.5 min. The total analysis time was 16.158 min and the gas flow rate was 1 mL/min, with nitrogen as the carrier gas. Qualitative and quantitative analysis were done using ChemStation B.01.04 specialist software (Agilent Technologies, Cheadle, UK). Fatty acids were identified on the basis of retention times predetermined for the respective standards (Sigma-Aldrich and Neochema, Cayman Islands) used in the method. The results were normalized using the internal standard C21, added to each test during isolation. The results are presented as the percentage of the individual fatty acids in the total mass of fatty acids from the examined tissue (internal standard excluded).

### 2.4. Statistical Analyses

The results were statistically analyzed using R version 3.4.1 (RStudio, Boston, MA, USA) The normality of the variable distribution was examined using the Shapiro–Wilk test. Consequently, continuous variables were expressed as medians with interquartile ranges. For qualitative variables, a number was given, also expressed as a percentage. Differences in sociodemographic and anthropometric variables, as well as in acid concentrations, were analyzed for the MHT categories using the Mann–Whitney test [47]. The significance level was assumed to be 0.05. To control for type I errors, the false discovery rate (FDR) approach was used. These calculations were performed using the p.adjust function of the stats package in R (R Foundation for Statistical Computing, Vienna, Austria, https://cran.r-project.org).

## 3. Results

A total of 156 women with a mean age of 60 years participated in this study and their characteristics are presented in Table 1. We observed no statistically significant differences in the sociodemographic, anthropometric, and clinical parameters among the women in terms of MHT usage.

### Fatty Acid Content

After determining the percentage of fatty acids for the women, we found that the most prevalent were palmitic acid C16:0 (24.61%), linoleic acid C18:2n-6 (18.25%), omega-6 fatty acid n-6 (27.78%) and saturated fatty acid SFA (46.49%). Of the isolated acids, negligible amounts of caprylic acid C8:0 (0%), stearidonic acid C18.4 (0%), erucic acid C22.1cis13 (0%), and cerolic acid C26.0 (0%) were found. These fatty acids were not considered for further analysis.

In the subsequent stage of the study, the association between the use of MHT and the concentration of saturated fatty acids was assessed. We found that women taking MHT had significantly higher (*p* < 0.05) concentrations of tetradecanoic acid (C14:0) and palmitic acid (C16:0) than that of the women not taking MHT. A statistical tendency (*p* = 0.053) toward lower levels of oleic acid (C18:1n-9) and arachidonic acid (C20:4) was seen. The results are presented in Table 2.

In the next stage of analysis, the effect of MHT on the concentration of omega-3 fatty acids (n-3), omega-6 fatty acids (n-6), omega-9 fatty acids (n-9), unsaturated fatty acids (UFA), saturated fatty acids (SFA), monounsaturated fatty acids (MUFA), polyunsaturated fatty acids (PUFA), as well as the n-PUFA:SFA, MUFA:SFA, UFA:SFA, and n-6:n-3 ratios were estimated. The fatty acid content was calculated as follows: sum of n-3 = C18:3n-3 + C20:5 + C22:5n-3 + C22:6n-3, sum of n-6 = C18:2n-6 + C18:3n-6 + C20:4 + C22:4n-6, saturated fatty acids SFAs = C10:0 + C12:0 + C14:0 + C15:0 + C16:0 + C17:0 + C18:0 + C22:0 + C23:0, MUFAs = C14:1 + 16:1 + C18:1n-9 + C18:1trans11 + C24:1, PUFAs = 18:2n-6 + C18:3n-6 + C18:3n-3 + C20:4 + C20:5 + C22:4n-6 + C22:5n-3 + C22:6n-3. Unsaturated fatty acids (UFAs) were the sum of the MUFAs and PUFAs.

It was demonstrated that women using MHT had significantly lower (*p* < 0.05) concentrations of all these, with the exception of SFA (Table 3).

In our next step, we correlated the concentration of each fatty acid with the duration of MHT. We found a weak negative correlation and a weak positive correlation for linoleic acid (C18:2n-6) and nervonic acid (C24:1), respectively. The correlation coefficients are shown in Table 4.

In the final step of the analysis, we calculated the following fatty acid indices associated with cardiovascular disease: 16:1n-7/16:0 (delta-9 desaturase index), 18:0/18:2n-6 (delta-6 desaturase index), and eicosapentaenoic acid + docosahexaenoic acid (EPA + DHA, omega-3 index). We did not find any of these to be significantly linked with MHT. We also did not find any correlation between desaturase indices and the omega-3 index and the duration of MHT (*p* > 0.05), as evaluated using Spearman correlation analysis (Table 5).

## 4. Discussion

Menopause typically overlaps with the natural aging process in women. It has been elegantly demonstrated that climacteric (and thus age-related) physiological and biochemical malfunctions elevate the risk of a number of clinical entities, among them cardiovascular and mood-related problems. The mechanism can be associated with a skewed lipid profile and, at least partly, explained by estrogen deficiency [31,35]. Some biotherapeutics (such as isoflavones, prenylflavonoids, and bovine colostrum) that possess demonstrated efficacy in diminishing the vulvovaginal atrophy and vasomotor symptoms of menopause do exist and have been shown not to elevate the risk of metabolic malfunctions [48]. We aimed to find the relationship between MHT and fatty acid profile in postmenopausal women. We found that, in females receiving sex hormones, the concentration of C14:0 and C16:0 was elevated, whereas the levels of C18:1n-9 and C20:4 decreased as compared with women not receiving MHT. Hormone therapy significantly decreased the content of all saturated fatty acids. The concentration of SFA was significantly elevated in the women receiving MHT, and there were no differences in n-3 concentration between subgroups. The longer the duration of MHT, the higher the concentration of C24:1 and the lower the concentration of C18:2n-6 found.

There have been a few studies evaluating fatty acids levels. We were able to replicate some of the findings from these observational trials. Somewhat similar results were reported by Stark et al. [49], who determined the concentration of serum fatty acids in 54 women and found that all women using MHT had significantly higher levels of C16:0, C16:1, and C20:3n-6, and significantly lower levels of C22:0, C24:0, C24:1 than that of the women not receiving hormones, underlining the negative impact of MHT on lipid profile. Similarly, Ottosson et al. [50] examined the effect of oral estrogen administration in 48 postmenopausal women and found that ethinylestradiol treatment increased C16:0 levels and decreased C18:0 content. They also demonstrated an increased risk of myocardial infarction and stroke due to the high levels of C20:3n-6 and the low DPA in the studied women. Sumino et al. [51] investigated the effect of MHT on the concentration of DPA and EPA in 104 postmenopausal women who took either estrogen or medroxyprogesterone for twelve months. There was a significant increase in DPA and EPA in the women taking hormones, which might have had a significant antiatherosclerotic effect on these postmenopausal women. Similarly, Piperi et al. [52] investigated the level of major fatty acids and phospholipids in 127 women aged 46 to 68 years and found a significant reduction in C15:0 and C20:0, along with an increase in oleic and linoleic acid in postmenopausal women receiving both estrogen and progestogens as compared with single estrogen therapy. However, Giltay et al. [53] studied the effect of hormone replacement therapy and raloxifene, finding that 150 mg of raloxifene and HRT increased the levels of C22:6n-3 and C20:4n-6 in the plasma of their subjects.

Similar to some other authors, we have demonstrated that MHT elevates particular SFAs and negatively affects the concentration of UFA, however, a skewed lipid profile may be present during aging process, independent of hormone use. This was suggested by a 2016 study [54] of 102 premenopausal women with iron deficiency anemia and 88 healthy women, the results of which demonstrated that, in both groups, the most prevalent fatty acids were palmitic acid, oleic acid, linoleic acid, stearic acid, and erucic acid. Such age-dependent alterations in fatty acid profiles have been documented before [55]. As shown in a few studies, an increased concentration of palmitic acid, palmitoleic acid, and dihomo-g-linolenic acid, along with low proportions of LA, may increase the risk of cardiometabolic entities [56], including myocardial infarction [57], stroke [58], left ventricular hypertrophy, and metabolic syndrome [59,60]. In particular, an elevated content of palmitic acid has been associated with the creation of carotid plaque and skewed carbohydrate metabolism, as well as hypertension [61]. For instance, Kim et al. [62] examined FA levels in 55 middle-aged women and found significant increases in the C14:0 and C16:0 content of erythrocyte membranes in people with increased triglycerides and blood pressure. Although a meta-analysis looking for the association between SFA intake and cardiovascular well-being found no evident linkage, study bias may have influenced these results [63], and therefore the debate continues.

Increased intake of UFA was found to significantly decrease the risk of CVD [64,65]. It is worth noting that the women in our study group who took MHT had low levels of C18:1n-9 and C20:4 and overall significantly lowered concentrations of MUFA and PUFA. As demonstrated by Bolton-Smith et al., the aging process itself requires UFAs supplementation [55]. This is essential, as it has been demonstrated that replacement of SFA by isocaloric MUFA improves lipid profile, in particular lowering the concentration of LDL while maintaining HDL content [66,67]. In comparison to a carbohydrate diet, meals with high MUFA content have also been shown to have a favorable effect on total cholesterol (TC) concentration and triglyceride (TAG) levels [68,69,70]. Although the effects of MUFAs and PUFAs on TC and TAG are comparable, MUFAs have shown have more beneficial effects on the HDL level [71]. A recent study has also found that, in patients with nonalcoholic fatty liver disease, caloric restriction may significantly diminish SFA levels and increase PUFA concentrations [72], which underlines how the ingestion of certain fatty acids may independently be (aside from MHT) responsible for the unfavorable changes in FA profile in our study group. Stark et al. [70] examined 38 postmenopausal women and assessed the effect of DHA supplementation on the risk of cardiovascular disease; almost half of these women took HRT. It was observed that SFA constituted 44.40%, MUFA 15.32%, PUFA 40.29%, n-6 34.05%, and n-3 6.23%, while the DHA:EPA ratio was 4.06. In the case of DHA supplementation, the accumulation of EPA in serum and in phospholipids was significantly more attenuated in postmenopausal women taking HRT than that of women without hormonal treatment.

The decrease in EPA and DHA, in our study group, negatively affected the omega-3 index parameter (though insignificantly), which has previously been found to predict CVD manifestations. Von Schacky et al. reviewed the value of the omega-3 index in various populations, finding that in America it equaled 4.9%, in Germany 5.6%, in Japan 8.5% and in Korea 11% [71]. In our study, the value of these indices in women treated with MHT was below the recommended level of 4%, however, the controls showed values slightly above this cut off, which makes the whole study group more prone to develop CVD. This could, at least partly, be explained by the elevated ingestion of SFA [72] and the mediation of an estrogen-dependent mechanism [39]. We found that the values of the delta-6 and delta-9 desaturase indices were not dependent on MHT therapy and were insignificantly elevated in the controls.

In this study, we analyzed fatty acids in whole blood. The choice of this method was determined by the assessment of morphotic parameters and the long-term response of the body. The study by Risé et al. [73] confirmed that the fatty acid profile in the whole blood reflects a balanced proportion of fatty acids in the plasma and blood cells. Furthermore, the study by Marangoni et al. [74] demonstrated that the content of fatty acids in the whole blood, plasma, and capillary blood is similar. In their meta-analysis, Stark et al. [75] demonstrated that the levels of fatty acids were mainly determined in plasma total lipids, plasma phospholipids, erythrocytes, and whole blood. Stark et al. emphasized that there is no “gold standard” in the measurement of fatty acids, which causes difficulties in comparing studies performed throughout the world. In addition, many scientists may have problems using erythrocytes to determine the levels of fatty acids due to logistic challenges associated with preparation and storing the samples [76,77]. While the fatty acid profile in the whole blood and the fatty acid profile in the plasma are very much alike, whole blood analysis is a faster, widely available, and easy-to-use method.

## 5. Conclusions

The present study demonstrates the negative effect of menopausal hormonal therapy on the FA profile, elevating saturated fatty acids (SFA,) and diminishing unsaturated fatty acids (UFA), in a study of women.

## 6. Limitations

The level of FA was measured in the whole blood, not in the erythrocytes. The study sample consisted of 171 women, and a greater number of participants would have strengthened the study. Unfortunately, we did not manage to find a greater number of women who met the inclusion criteria. Other aspects that could be regarded as limitations include the recruitment method (posters and advertisements), and the fact that recruitment was restricted to one voivodeship (province), which made it impossible for us to reach a wider group of potential participants.

## Figures and Tables

**Table 1:** Patient characteristics.

Variables	Women Using MHT *n* = 49			Women Not Using MHT *n* = 107			All Women *n* = 156		*p*	FDR
	Me	IQR	Average Rank	Me	IQR	Average Rank	Me	IQR		
Age (y)	57	7	64.5	60	8	84.9	59	7.8	0.009	0.045
BMI (kg/m^2^)	25.7	6.2	69.3	26.6	6.0	82.7	26.1	6.0	0.087	0.162
Height (cm)	164	8	92.3	160	7	72.2	162	9	0.0099	0.045
Body weight (kg)	70	17	76.4	70	17	79.5	70	16.5	0.697	0.690
WHR	0.8	0.09	71.5	0.8	0.08	81.7	0.8	0.07	0.189	0.162
Waist (cm)	84	14	68.4	86	16	83.1	85	15	0.06	0.162
Hips (cm)	104	12.5	74.8	104	12	80.2	104	12.5	0.486	0.551
Age of first menstruation [y]	14	2	83.1	14	2	76.4	14	2	0.376	0.551
Age of menopause [y]	50	3.5	74.5	51	5	80.3	51	5	0.457	0.551

BMI: body mass index; WHR: waist-hip ratio; Me: median; IQR: interquartile range; FDR: false discovery rate.

**Table 2:** Concentrations of fatty acids in postmenopausal women in terms of menopausal hormonal therapy (MHT) using the Mann–Whitney test.

Fatty Acid (%)		Women Using MHT *n* = 49			Women Not Using MHT *n* = 107			*p*	FDR
		Me	IQR	Average Rank	Me	IQR	Average Rank		
C10:0	decanoic acid	1.74	1.19	79.8	1.62	1.35	77.9	0.810	0.836
C12:0	dodecanoic acid	0.25	0.12	83.2	0.24	0.1	76.3	0.374	0.569
C14:0	tetradecanoic acid	1.47	0.64	95.6	1.15	0.74	70.7	0.001	0.011
C14:1	myristoleic acid	0.1	0.12	75.5	0.12	0.11	79.9	0.577	0.673
C15:0	pentadecanoic acid	0.33	0.23	75.5	0.34	0.17	79.9	0.578	0.673
C16:0	palmitic acid	26.44	4.12	98.8	22.89	5.0	69.2	0.0001	0.001
C16:1	palmitoleic acid	1.27	0.64	77.5	1.24	0.55	79.0	0.849	0.852
C17:0	heptadecanoic acid	0.42	0.12	82.7	0.41	0.11	76.6	0.431	0.606
C18:0	stearic acid	20.90	13.51	87.3	11.7	12.34	74.5	0.100	0.242
C18:1n-9	oleic acid (OA)	14.65	4.26	64.8	17.23	4.59	84.8	0.010	0.055
C18:1trans11	trans-vaccenic acid (VA)	1.38	0.63	70.2	1.58	0.55	82.3	0.119	0.242
C18:2n-6	linoleic acid (LA)	16.91	5.54	69.2	18.84	6.69	82.8	0.081	0.220
C18:3n-6	gamma linolenic acid (GLA)	0.21	0.19	75.7	0.29	0.17	79.8	0.612	0.673
C18:3n-3	α linolenic acid (ALA)	0.56	0.23	67.2	0.58	0.26	79.8	0.602	0.124
C20:4	arachidonic acid (ARA)	7.08	2.92	64.4	8.58	3.2	84.9	0.009	0.055
C20:5	eicosapentaenoic acid (EPA)	1.14	0.49	75.3	1.22	0.9	80.0	0.552	0.673
C22:0	behenic acid	0.00	0.37	67.6	0.31	0.48	83.5	0.034	0.124
C22:4n-6	docosatetraenoic acid (DTA)	0.75	0.36	72.3	0.81	0.41	81.4	0.243	0.422
C22:5n-3	docosapentaenoic acid (DPA)	1.20	0.48	72.4	1.26	0.58	81.3	0.250	0.422
C22:6n-3	docosahexaenoic acid (DHA)	2.47	1.03	73.8	2.72	1.4	80.6	0.388	0.569
C23:0	tricosanoic acid	0.12	0.25	71.6	0.17	0.29	81.7	0.187	0.374
C24:1	nervonic acid	0.00	0.00	70.6	0.00	0.14	82.1	0.042	0.135

Me: median; IQR: interquartile range; FDR: false discovery rate.

**Table 3:** Fatty acid profiles of postmenopausal women in terms of MHT usage using the Mann–Whitney test.

Fatty Acid (%)	Women Using MHT *n* = 49		Women Not Using MHT *n* = 107		*p*	FDR
	Me	IQR	Me	IQR		
n-3	5.43	1.91	5.86	2.92	0.325	0.036
n-6	24.66	9.04	29.18	10.15	0.034	0.047
n-9	14.65	4.47	17.38	4.54	0.01	0.018
UFA	47.61	16.63	59.47	16.65	0.005	0.018
SFA	52.39	16.63	40.53	16.65	0.005	0.018
MUFA	16.17	4.81	18.82	5.38	0.013	0.020
PUFA	30.82	10.98	37.22	12.41	0.041	0.050
PUFA:SFA	0.56	0.49	0.92	0.53	0.01	0.018
MUFA:SFA	0.34	0.24	0.47	0.26	0.007	0.018
UFA:SFA	0.91	0.74	1.47	0.82	0.005	0.018
n-6:n-3	4.6	1.59	4.63	1.64	0.764	0.760

Me: median; IQR: interquartile range; FDR: false discovery rate; n-3: omega-3 fatty acids; n-6: omega-6 fatty acids; n-9: omega-9 fatty acids; UFA: unsaturated fatty acids; SFA: saturated fatty acids; MUFA: monounsaturated fatty acids; PUFA: polyunsaturated fatty acids.

**Table 4:** Correlation between FA concentration and the duration of MHT (years).

Fatty Acid (%), *n* = 156		r	*p*	FDR
C10:0	decanoic acid	0.073	0.36	0.98
C12:0	dodecanoic acid	0.081	0.31	0.98
C14:0	tetradecanoic acid	0.002	0.98	0.98
C14:1	myristoleic acid	0.036	0.66	0.98
C15:0	pentadecanoic acid	0.054	0.50	0.98
C16:0	palmitic acid	−0.051	0.53	0.98
C16:1	palmitoleic acid	−0.085	0.29	0.98
C17:0	heptadecanoic acid	0.05	0.56	0.98
C18:0	stearic acid	0.04	0.62	0.98
C18:1n-9	oleic acid (OA)	0.05	0.56	0.98
C18:1trans11	trans-vaccenic acid (VA)	0.008	0.94	0.98
C18:2n-6	linoleic acid (LA)	−0.17	0.03	0.33
C18:3n-6	gamma linolenic acid (GLA)	−0.008	0.92	0.98
C18:3n-3	α linolenic acid (ALA)	−0.13	0.11	0.81
C20:4	arachidonic acid (ARA)	0.03	0.70	0.98
C20:5	eicosapentaenoic acid (EPA)	−0.002	0.98	0.98
C22:0	behenic acid	0.053	0.91	0.98
C22:4n-6	docosatetraenoic acid (DTA)	0.013	0.86	0.98
C22:5n-3	docosapentaenoic acid (DPA)	0.076	0.35	0.98
C22:6n-3	docosahexaenoic acid (DHA)	−0.075	0.35	0.98
C23:0	tricosanoic acid	−0.007	0.93	0.98
C24:1	nervonic acid	0.234	0.004	0.09

r: correlation coefficient; *p*: significance level; FDR: false discovery rate.

**Table 5:** Correlation between desaturase and omega-3 indices and the duration of MHT (years).

Fatty Acid Index	Women Using MHT *n* = 49		Women Not Using MHT *n* = 107		*p*	FDR
	Me	IQR	Me	IQR		
16:1n-7/16:0 (D9D)	0.05	0.03	0.06	0.03	0.27	0.41
18:0/18:2n-6 (D6D)	0.013	0.012	0.015	0.01	0.12	0.36
EPA + DHA (omega-3 index)	3.74	1.71	4.01	2.33	0.57	0.57

D6D: delta-6 desaturase; D9D: delta-9 desaturase; Me: median; IQR: interquartile range; FDR: false discovery rate.
